# Hypoxia-inducible factor 1 alpha protein increases without changes in mRNA during acute hypoxic exposure of the Gulf killifish, *Fundulus grandis*

**DOI:** 10.1242/bio.060167

**Published:** 2023-12-27

**Authors:** Taylor E. Murphy, Jasmine C. Harris, Bernard B. Rees

**Affiliations:** Department of Biological Sciences, University of New Orleans, New Orleans, LA, 70148, USA

**Keywords:** Oxygen, Teleost fish, Transcription factor, Individual variation

## Abstract

The hypoxia inducible factor 1 (HIF1) is a central regulator of the molecular responses of animals to low oxygen. While the hypoxia-responsiveness of HIF1 is generally attributed to the stabilization of the alpha protein subunit (HIF1α) at low oxygen, several studies on fish report increased tissue levels of *HIF1A* mRNA during hypoxia, suggesting transcriptional regulation. In the current study, HIF1α protein and *HIF1A* mRNA were determined in parallel in tissues of Gulf killifish, *Fundulus grandis*, exposed to short-term hypoxia (24 h at 1 mg O_2_ l^−1^). HIF1α protein was higher in brain, ovary, and skeletal muscle from fish exposed to hypoxia compared with normoxic controls by 6 h, and it remained elevated in brain and ovary at 24 h. In contrast, *HIF1A* mRNA levels were unaffected by hypoxia in any tissue. Moreover, HIF1α protein and *HIF1A* mRNA levels in the same tissues were not correlated with one another during either normoxia or hypoxia. Hence, an increase in HIF1α protein does not depend upon an increase in *HIF1A* mRNA during acute exposure to low oxygen in this species. The results support the widely accepted mechanism of post-translational protein stabilization, rather than new transcription, during the initial response of fish to hypoxia.

## Teaser

**Summary:** Parallel measurements of hypoxia inducible factor protein and mRNA support post-translational protein stabilization rather than new transcription in the initial response of fish to low oxygen.

## INTRODUCTION

Although low dissolved oxygen (hypoxia) is a naturally occurring phenomenon in aquatic environments, its geographic scope and severity have increased in recent decades (Jenny et al., 2016; Breitburg et al., 2018). Because oxygen is critical for aerobic energy metabolism, decreased levels of oxygen have wide-ranging and frequently dramatic biological effects, including changes in behavior, impaired growth and reproduction, and increased mortality, all of which may contribute to changes in biodiversity in aquatic habitats (Vaquer-Sunyer and Duarte, 2008; Small et al., 2014; Jenny et al., 2016; Breitburg et al., 2018). When exposed to sub-lethal levels of hypoxia, many species of fish show changes in gene expression, which may improve their ability to tolerate low oxygen (Richards, 2009). Thus, hypoxia-tolerant fishes are valuable models for assessing the molecular responses of animals to hypoxia (Nikinmaa and Rees, 2005; Mandic et al., 2009; Richards, 2009), which may help determine their resilience in the current context of changing aquatic habitats.

The hypoxia-inducible transcription factors (HIFs) are evolutionarily conserved, central regulators of the molecular responses to low oxygen in animals (Semenza, 2009, 2012; Majmundar et al., 2010; Pamenter et al., 2020; Mandic et al., 2021; Townley et al., 2022). HIF1 was first identified in mammalian cell culture as a protein required for the hypoxic induction of the glycoprotein hormone, erythropoietin (Semenza and Wang, 1992; Maxwell et al., 1993). The active transcription factor is a dimer composed of HIF1α and HIF1β subunits, the latter of which was previously described as the aryl hydrocarbon nuclear translocator (ARNT) (Wang et al., 1995; McIntosh et al., 2010). The oxygen dependency of HIF signaling is due, in part, to post-translational regulation of HIF1α protein concentration. During normoxia, prolyl hydroxylase domain (PHD) enzymes hydroxylate HIF1α at conserved proline residues, a process that targets the alpha subunit for ubiquitin-dependent proteasomal degradation. At low oxygen levels, PHD activity decreases, HIF1α degradation is suppressed, and HIF1α protein subunits accumulate (Kaelin and Ratcliffe, 2008). HIF1α dimerizes with ARNT, translocates to the nucleus, binds to specific DNA-regulatory elements in target genes, and promotes transcription. HIF1 regulates the expression of over 100 genes, many of which improve oxygen delivery to tissues or improve the capacity of tissues to tolerate low oxygen (Wenger et al., 2005; Ortiz-Barahona et al., 2010; Semenza, 2009, 2012). Although an increase in HIF1α subunit stability is widely-accepted as the primary mechanism leading to elevated levels of this transcription factor during hypoxia, some studies have reported increased *HIF1A* mRNA levels (e.g. Bergeron et al., 1999, but see Stroka et al., 2001), increased rates of HIF1α synthesis (Semenza, 2009), and other post-translational modifications (Albanese et al., 2020), which may contribute to HIF1 stability or activity.

Soitamo et al. (2001) were the first to report the presence of HIF1α in fish. They sequenced HIF1α from rainbow trout (*Oncorhynchus mykiss*) and showed that HIF1α protein increased in abundance during short-term (4 h) hypoxic exposure of salmonid liver, gonad, and embryonic cells in culture. Two important observations were that the increase in HIF1α protein during hypoxia occurred when mRNA synthesis was inhibited, and that pharmacological inhibition of the proteasome during normoxia resulted in an increase in HIF1α protein. These observations suggested that HIF1α protein abundance in fish was post-translationally regulated in a similar fashion to that described in mammals. Subsequently, hypoxia-induced elevation of HIF1α protein has been reported in fish embryos, cultured cells, and adult tissues (Mandic et al., 2021 and references therein), although the extent of this elevation depends on experimental conditions (level and duration of hypoxia; temperature), species, tissue, and individual (e.g. Rissanen et al., 2006).

Although Soitamo et al. (2001) showed that new transcription was not required for the hypoxia-dependent increases in HIF1α protein of salmonid cells in culture, several studies have reported elevated *HIF1A* mRNA levels in tissues of fish exposed to low oxygen (Mandic et al., 2021 and references therein). These studies vary in species, tissues, exposure conditions (e.g. depth and duration of hypoxia), and experimental detail (Bustin et al., 2009), making it difficult to ascertain the generality and biological relevance of changes in *HIF1A* mRNA. Further complicating the interpretation of these studies is the fact that certain fish lineages (e.g. Otocephala, which includes herring, catfish, carp, and zebrafish) retain both paralogs of *HIF1A* arising from a teleost-specific genome duplication, while more advanced fishes (Euteleost) have retained only a single paralog (Rytkönen et al., 2013; Townley et al., 2022). A comparison of the nucleotide sequences of PCR primers or amplification products shows that, more often than not, studies of Otocephala measured transcript levels of the teleost-specific paralog (*HIF1Ab*) different from the one found in Euteleost (T. E. Murphy, personal observations). Finally, data on both HIF1α protein and its corresponding mRNA are rarely reported from the same tissues of the same species, making it difficult to determine the contribution of transcriptional versus post-translational regulation of HIF1 during the response of fish to hypoxia.

In this study, therefore, we examined the relationship between levels of HIF1α protein and *HIF1A* mRNA in tissues of the Gulf killifish *Fundulus grandis*, Baird and Girard 1853, exposed to acute hypoxia. *Fundulus grandis* is a widespread and ecologically important species of the salt marsh communities of the Gulf of Mexico (Nordlie, 2006), areas that are prone to natural and anthropogenic hypoxia (Engle et al., 1999). This species is in the order Cyprinidontiformes, which is part of the Euteleost (Hughes et al., 2018), thus possessing a single *HIF1A* paralog. Comparison of genomic sequences and the order of nearby genes shows that the gene retained in *F. grandis* and other Euteleost is more closely related to *HIF1Aa* than *HIF1Ab* in the Otocephala, which retain both paralogs (Rytkönen et al., 2013; Townley et al., 2022). Previous research showed that HIF1α protein increased in several tissues of *F. grandis* exposed to 1 mg O_2_ l^−1^ (∼13% of the air-saturated oxygen concentration) for 24 h (Gonzalez-Rosario, 2016). Here, we asked whether the increase in HIF1α protein, determined by immunoprecipitation and Western blotting, was associated with an increase in *HIF1A* mRNA levels, determined by quantitative PCR, when measured in the same tissues.

Furthermore, *HIF1A* is part of a larger gene family that includes *HIF2A*, *HIF3A*, and *HIF4A* (Rytkönen et al., 2011; Graham and Presnell, 2017; Townley et al., 2022). Most fish lineages have retained both teleost-specific duplicates of *HIF2A*, although one form is greatly truncated in the Euteleost (Rytkönen et al., 2013; Townley et al., 2022). The form that encodes a full-length protein was first described in *F. heteroclitus* (Powell and Hahn, 2002) and it has been designated as *HIF2Aa* (Rytkönen et al., 2013), a convention we follow here, although it has been referred to as endothelial PAS-domain protein 1b (*EPAS1b*) or *HIF2Ab* in many databases. Most Euteleost, including *F. grandis*, appear to have only one form of *HIF3A* and they lack *HIF4A* entirely (Townley et al., 2022). Thus, we also measured the mRNA abundance of *HIF2Aa* and *HIF3A* in tissues of normoxic and hypoxic *F. grandis*.

## RESULTS

### Indicators of hypoxia exposure

**Fig. 1.**
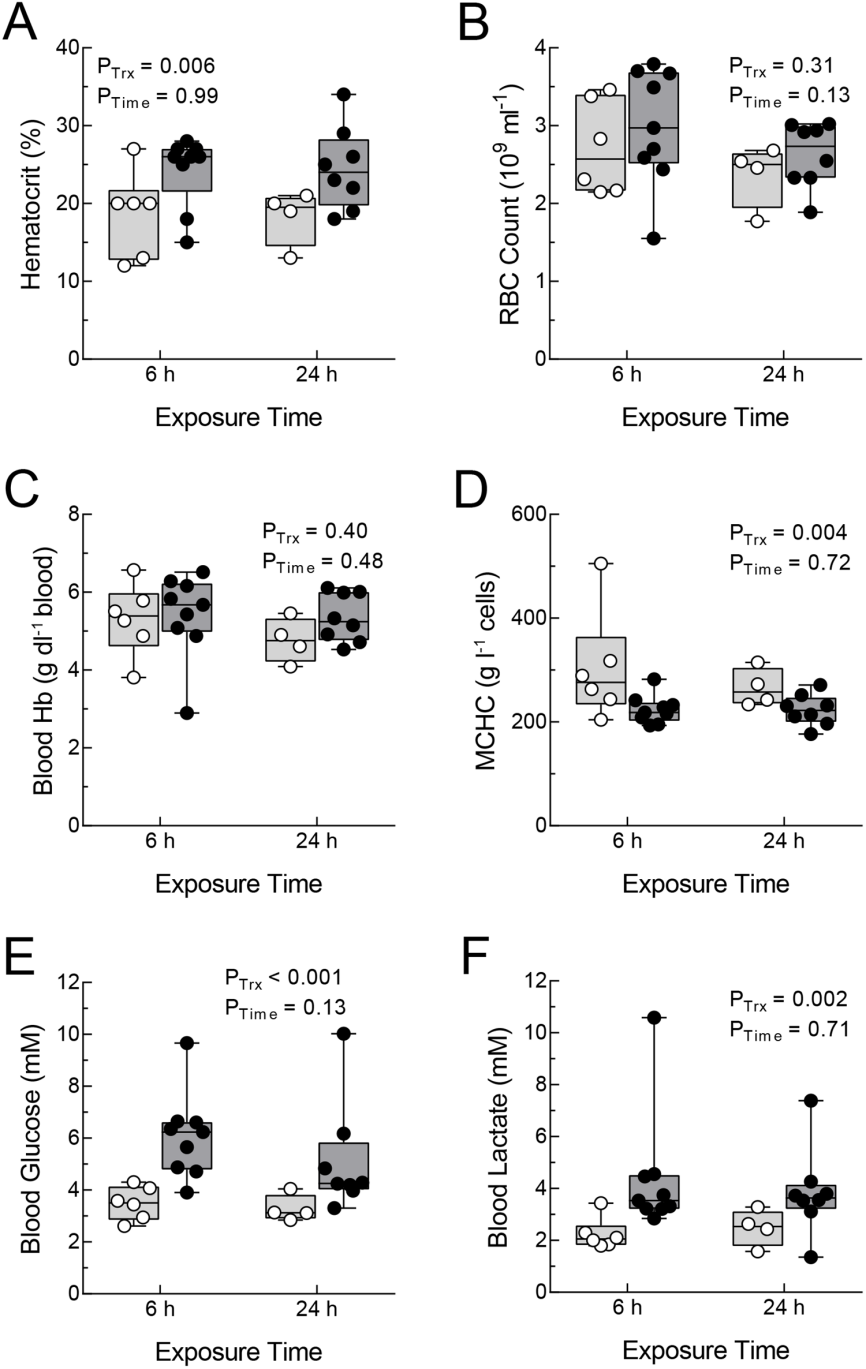
**Blood indicators of oxygen transport and carbohydrate metabolism in *Fundulus grandis* during acute hypoxia.** Fish were exposed to normoxia (>7 mg O_2_ l^−1^; light grey boxes and open symbols) or hypoxia (∼1 mg O_2_ l^−1^; dark grey boxes and filled symbols) for 6 or 24 h and sampled for hematocrit (A), red blood cell (RBC) count (B), blood hemoglobin (Hb) (C), mean corpuscular hemoglobin concentration (MCHC) (D), blood glucose (E), and blood lactate (F). Lines of box and whiskers plots represent (from lowest to highest) the minimum, the 25th percentile, the median, the 75th percentile, and the maximum. The effects of hypoxia treatment (*P*_Trx_), exposure time (*P*_Time_), and the interaction between treatment and time were determined by two-way ANOVA (all interactions were non-significant and not shown).

Blood variables reflecting oxygen transport capacity and carbohydrate metabolism were measured in *F. grandis* to verify that the experimental exposures caused oxygen limitation (Fig. 1). Exposure of fish to ∼1 mg O_2_ l^−1^ resulted in an increase in hematocrit (Fig. 1A), as expected. Although there was a trend toward increased red blood cell count, this change was not significant (Fig. 1B), suggesting that the major determinant of higher hematocrit during acute hypoxia was an increase in mean corpuscular volume (MCV). Indeed, MCV calculated for hypoxic fish (83×10^−15^ l at 6 h and 93×10^−15^ l at 24 h) was significantly greater (two-way ANOVA, p_Trx_=0.015) than that for normoxic fish (70×10^−15^ l at 6 h and 77×10^−15^ l at 24 h). Consistent with the lack of an effect of hypoxia on RBC count, there was no significant difference in blood hemoglobin (Hb) concentration between normoxic and hypoxic fish (Fig. 1C). Because hypoxia led to an increase in MCV with no increase in blood Hb, hypoxia caused a significant decrease in mean corpuscular hemoglobin concentration (MCHC) (Fig. 1D). Hypoxia also led to moderate hyperglycemia (Fig. 1E) and a significant increase in blood lactate (Fig. 1F). There was no effect of time of exposure, nor interactions between hypoxia treatment and exposure time, for any variable, indicating that these physiological responses to hypoxia occurred within the first 6 h of exposure and thereafter were unchanged for the duration of the experiment (24 h).

### HIF1α protein abundance

**Fig. 2.**
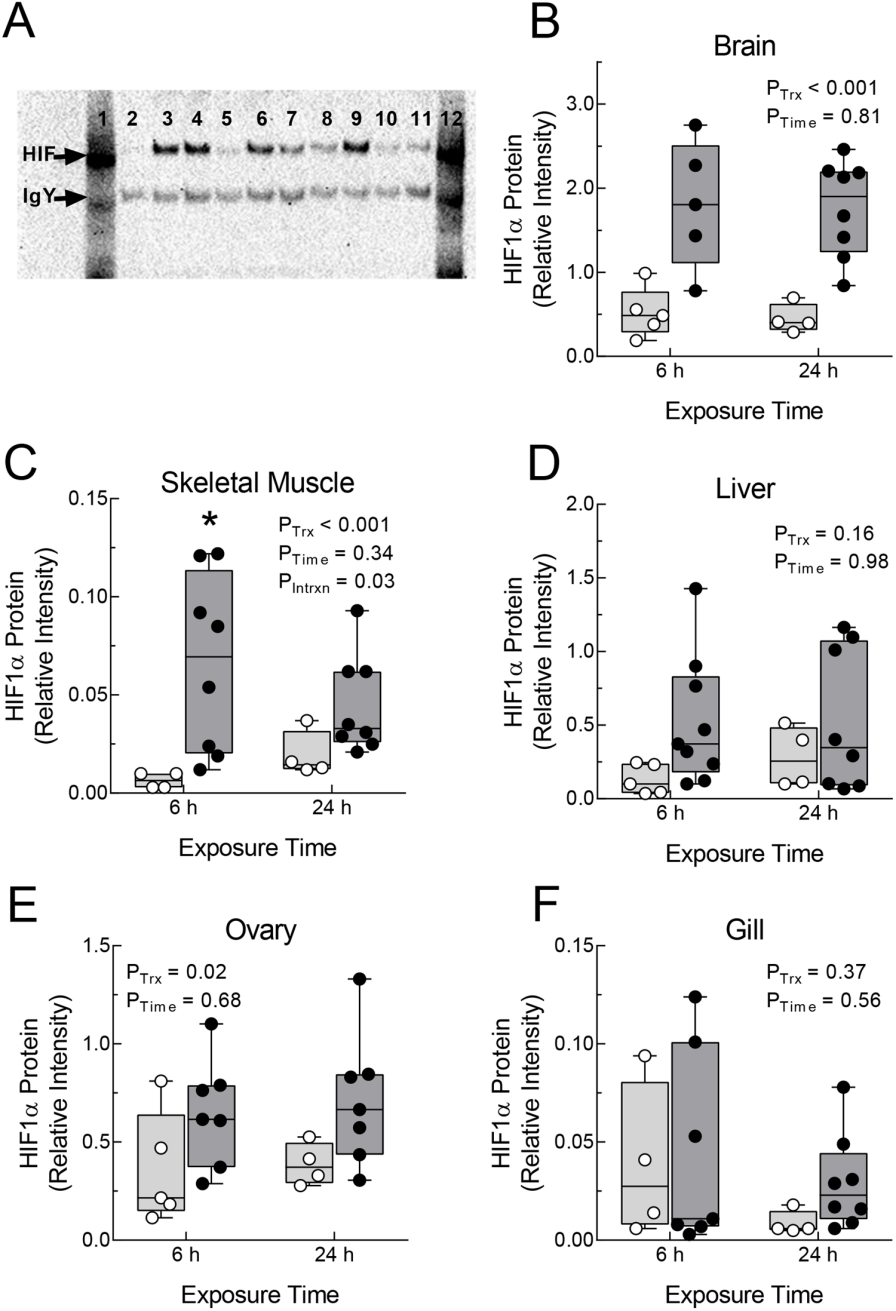
**HIF1α protein levels in tissues of *Fundulus grandis* during acute hypoxia.** Panel A shows a Western blot of HIF1α immunoprecipitated from brain lysates of fish exposed for 6 h to normoxia (>7 mg O_2_ l^−1^; lanes 2, 5, 8, 10, and 11) or hypoxia (∼1 mg O_2_ l^−1^; lanes 3-4, 6-7, and 9). A positive HIF1α control is shown in lane 1 and the mobility of HIF1α and chicken IgY are shown by arrows (at left). HIF1α protein abundance in each sample was expressed as the ratio of the HIF1α band intensity to the IgY band intensity. Relative HIF1α protein abundance was determined for brain (B), skeletal muscle (C), liver (D), ovary (E), and gill (F) after exposure of fish to normoxia (>7 mg O_2_ l^−1^; open symbols and light grey boxes) or hypoxia (∼1 mg O_2_ l^−1^; filled symbols and dark grey boxes) for 6 or 24 h. Lines of box and whiskers plots represent (from lowest to highest) the minimum, the 25th percentile, the median, the 75th percentile, and the maximum. The effects of hypoxia treatment (*P*_Trx_), exposure time (*P*_Time_), and the interaction between treatment and time were determined by two-way ANOVA. The treatment by time interaction was only significant for skeletal muscle, for which the effect of hypoxia on HIF1α abundance was significant at 6 h exposure (asterisk).

Levels of HIF1α protein, determined by immunoprecipitation followed by Western blotting, increased in *F. grandis* exposed to hypoxia in a tissue- and individual-dependent fashion (Fig. 2). An image of a Western blot of HIF1α protein immunoprecipitated from brain (Fig. 2A) shows that most of the hypoxic samples (lanes 3-4, 6-7, and 9) had higher levels of HIF1α protein than normoxic samples (lanes 2, 5, 8, and 10-11), although there was appreciable variability in HIF1α protein abundance in both hypoxic and normoxic fish. Some of this variation could be due to differences in the effectiveness of immunoprecipitation among samples. Hence, all subsequent analyses were performed on relative HIF1α abundance, calculated by dividing the HIF1α band intensity by the intensity of the immunoprecipitating IgY band for the same sample (see Materials and Methods).

The effects of hypoxia on relative HIF1α protein abundance were significant in brain (Fig. 2B) and ovary (Fig. 2E). In brain, HIF1α protein levels were about three times higher in hypoxia than in normoxia, and in ovaries, HIF1α protein levels were about twofold higher in hypoxia. In these two tissues, there was no effect of exposure time nor was there an interaction between hypoxia treatment and exposure time. In skeletal muscle, there was a significant interaction between treatment and exposure time, with significantly elevated HIF1α protein levels in hypoxic fish at 6 h compared to corresponding controls (Fig. 2C). For liver (Fig. 2D) and gill (Fig. 2F), there were no significant effects of treatment, exposure time, or their interaction. Of note, levels of HIF1α protein determined by immunoprecipitation were highest in brain, followed by ovary and liver. HIF1α protein levels were extremely low in skeletal muscle and gill during both normoxia and hypoxia.

Correlation analyses were conducted to assess whether HIF1α protein levels were consistently low or high across tissues among individuals within treatment groups (Table S1). After correction for multiple comparisons, levels of HIF1α protein were not correlated among tissues of either normoxic or hypoxic fish.

### *HIF1A* mRNA abundance

**Fig. 3.**
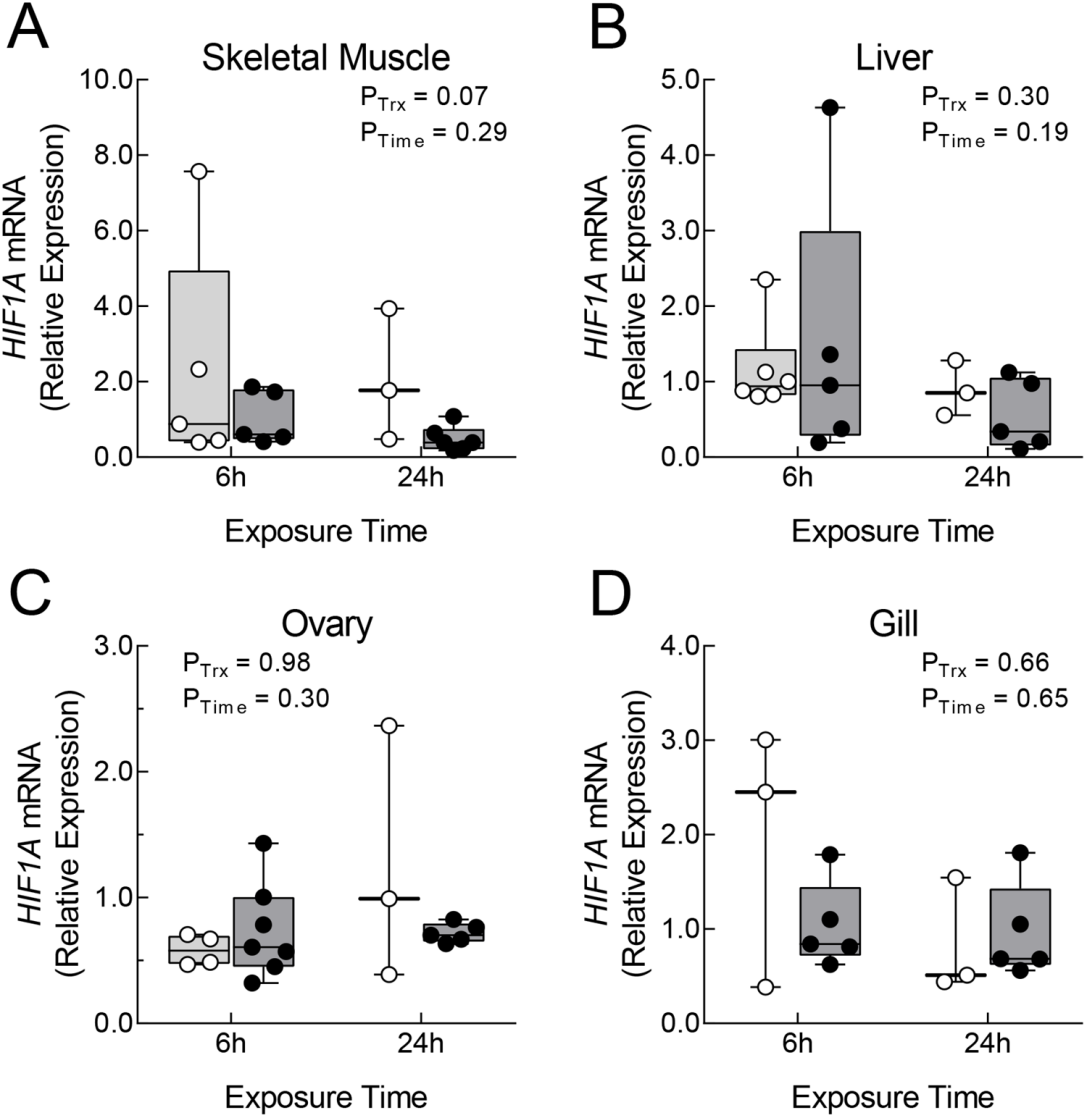
***HIF1A* mRNA levels in tissues of *Fundulus grandis* during acute hypoxia.** Levels of *HIF1A* mRNA were determined by qPCR and expressed relative to the level of *ARNT2* mRNA in the same samples. Relative *HIF1A* mRNA abundance was determined for skeletal muscle (A), liver (B), ovary (C), and gill (D) after exposure of fish to normoxia (>7 mg O_2_ l^−1^; open symbols and light grey boxes) or hypoxia (∼1 mg O_2_ l^−1^; filled symbols and dark grey boxes) for 6 or 24 h. Lines of box and whiskers plots represent (from lowest to highest) the minimum, the 25th percentile, the median, the 75th percentile, and the maximum. The effects of hypoxia treatment (*P*_Trx_), exposure time (*P*_Time_), and the interaction between treatment and time were determined by two-way ANOVA (all interactions were non-significant and not shown).

Levels of *HIF1A* mRNA were determined by qPCR in four tissues (the small size of brain tissue required that it be used exclusively for HIF1α protein determination). *HIF1A* mRNA levels were normalized by the levels of *ARNT2* mRNA, the transcript for the dimeric partner of HIF1α, which is not affected by hypoxic treatment (Rahman and Thomas, 2019; Table S3). Thus normalized, there were no significant effects of hypoxia treatment, exposure time, or their interaction on *HIF1A* mRNA levels in any tissue (Fig. 3). The only change that approached statistical significance was a trend toward lower levels of *HIF1A* mRNA in skeletal muscle during hypoxia (*P*_Trx_=0.07; Fig. 3A). As observed for HIF1α protein levels, correlation analyses showed that levels of *HIF1A* mRNA were not significantly related across tissues, in either normoxia or hypoxia (Table S2).

The copy number of *HIF1A* mRNA transcripts were determined from dilutions of known concentrations of a plasmid encoding *HIF1A* (Townley et al., 2017). When analyzed by two-way ANOVA, both the effects of hypoxia treatment and exposure time on gill *HIF1A* copy number approached statistical significance (0.10>*P*>0.05) (Table S3). However, these trends were mirrored by higher *ARNT2* in the same samples, suggesting that this effect was not specific to *HIF1A* (Table S3).

The levels of *HIF2Aa* and *HIF3A* mRNA were also determined in the same tissues. There were no effects of hypoxia treatment on *HIF2Aa* or *HIF3A* mRNA levels, either expressed relative to *ARNT2* mRNA levels (Figs S1 and S2) or as copy number (Table S3). Of note, the absolute level of *HIF2Aa* was the highest of all *HIFA* transcripts and it was particularly elevated in gill tissue (Table S3), as documented previously in *F. heteroclitus* and other fishes (Townley et al., 2017, 2022).

### Correlation between HIF1α protein and *HIF1A* mRNA

**Fig. 4.**
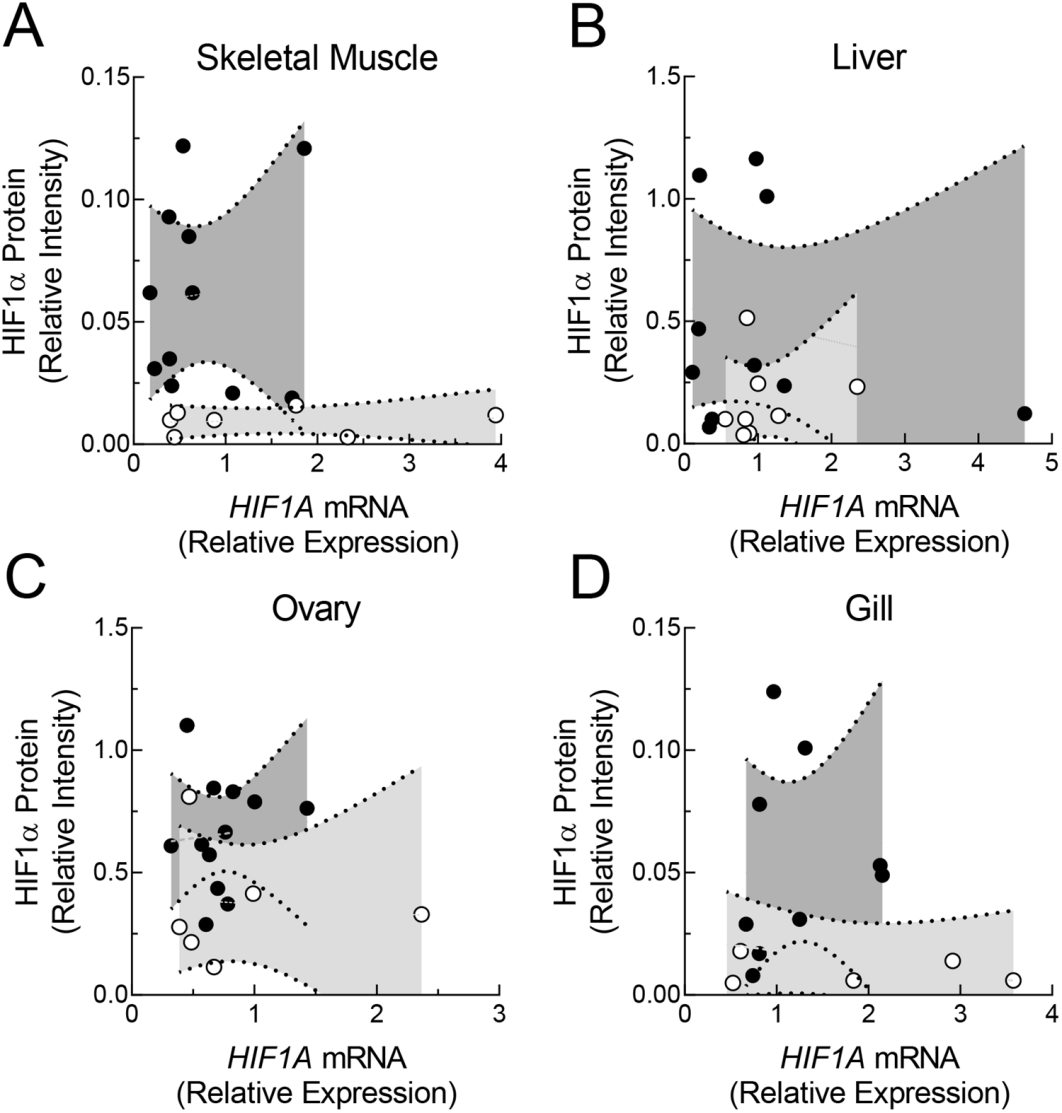
**The relationship between HIF1α protein and *HIF1A* mRNA levels in tissues of *Fundulus grandis* during acute hypoxia.** The relative abundance of HIF1α protein and *HIF1A* mRNA were determined in skeletal muscle (A), liver (B), ovary (C), and gill (D) and plotted against one another pooling time points (6 and 24 h) within treatment groups. Individual samples are shown as points and 95% confidence bands were determined by least squares linear regression of normoxic samples (open symbols and light grey confidence bands) and hypoxic samples (filled symbols and dark grey confidence bands).

The observations that HIF1α protein changes in certain tissues (Fig. 2) and *HIF1A* mRNA does not (Fig. 3) strongly argue that the levels of these two macromolecules are not linked during acute hypoxic exposure of *F. grandis*. Given that levels of HIF1α protein and *HIF1A* mRNA both displayed considerable variation (Figs 3 and 4), however, it was possible that their abundances covary among individuals within treatment groups, even if there were no apparent effects of hypoxia on the mean values. Thus, the correlations between tissue levels of HIF1α protein and *HIF1A* mRNA among individuals were examined in both treatment groups (Fig. 4). In none of the tissues examined was the correlation between HIF1α protein and *HIF1A* mRNA significant, during either normoxia or hypoxia (Table 1).

**Table 1.**
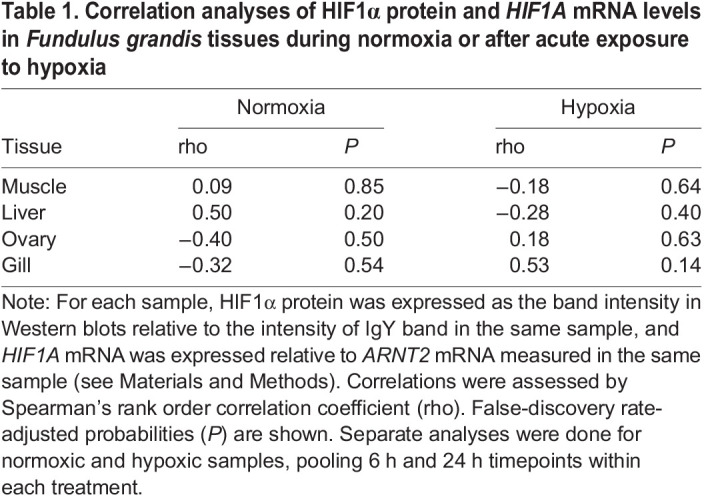
Correlation analyses of HIF1α protein and *HIF1A* mRNA levels in *Fundulus grandis* tissues during normoxia or after acute exposure to hypoxia

## DISCUSSION

### Validation of experimental exposure

The main goal of this study was to determine the levels of HIF1α protein and *HIF1A* mRNA in tissues of the Gulf killifish, *F. grandis*, when exposed to short-term hypoxia (6 or 24 h at 1 mg O_2_ l^−1^ or ∼13% of air-saturation at 25°C). These exposure conditions led to higher hematocrit, blood glucose, and blood lactate, all of which are typical responses of fish to low oxygen availability (Gallaugher and Farrell, 1998; Virani and Rees, 2000; Larter and Rees, 2017; Richards, 2009). The increase in hematocrit was mainly accounted for by RBC swelling, rather than an increase in RBC number. This increase in RBC volume was likely due to adrenergic stimulation of erythrocytic Na^+^-H^+^ exchange, which increases RBC pH and promotes osmotic swelling of cells (due to influx of Na^+^ balanced by Cl^−^ uptake via Cl-HCO_3_^−^ exchange) and serves to increase hemoglobin oxygen affinity (Nikinmaa and Salama, 1998). These changes indicated that the experimental conditions elicited physiological responses to low oxygen early (within 6 h) and that they persisted for the duration of the study (24 h).

### Increased HIF1α protein is an early response to acute hypoxia

Under these conditions, HIF1α protein was higher in brain, ovary, and skeletal muscle from fish exposed to hypoxia compared with normoxic controls by 6 h, and HIF1α protein remained elevated in brain and ovary at 24 h. Although there was a trend toward higher HIF1α protein in liver at both 6 and 24 h, this was not statistically significant due to high variability among individuals (see below). In an earlier study of *F. grandis* using similar experimental approaches, Gonzalez-Rosario (2016) showed that HIF1α protein increased in the same tissues after 24 h exposure of *F. grandis*, and Borowiec et al. (2018) reported higher levels in skeletal muscle after 12 h exposure of *F. heteroclitus*. Law et al. (2006) showed that exposure of grass carp to 0.5 mg O_2_ l^−1^ for 4 or 24 h increased HIF1α protein in liver, and Rissanen et al. (2006) reported that exposure of Crucian carp to 0.7 mg O_2_ l^−1^ for 6–48 h led to higher HIF1α in various tissues (liver, heart, gills, and kidney), although the magnitude of the increase differed among tissues and depended upon exposure duration and temperature. More recently, O'Brien et al. (2020) documented increased HIF1α protein in heart of Antarctic icefish, *Notothenia coriiceps*, after 12 h of hypoxia. Taken together, an increase in HIF1α protein in many tissues appears to be part of the early response of several fishes to acute hypoxic exposure. Interestingly, the increase in HIF1α in *F. grandis* brain was the largest in magnitude among tissues and least variable among individuals, perhaps indicating a more prominent role for this transcription factor in brain than in the other tissues examined.

In contrast, we found that HIF1α protein levels in *F. grandis* gill are very low and unaffected by hypoxia. This observation differs from that reported for Crucian carp, which displayed substantial levels of HIF1α during normoxia that were further increased by hypoxia (Sollid et al., 2006; Rissanen et al., 2006). The increase during hypoxia (0.7 mg O_2_ l^−1^) in Crucian carp gill depended upon temperature, occurring at 8°C, but not at 18°C or 26°C. Sollid et al. (2006) proposed that HIF1α protein plays a role in the remodeling of gills to increase the surface area for gas exchange during hypoxia (Sollid et al., 2003). Thus, it is possible that the difference in gill HIF1α protein abundance between Crucian carp and *F. grandis* correlates with their capacity for gill remodeling. Although this process has not been examined in *F. grandis*, it was not observed in the closely related *F. heteroclitus* during hypoxic exposures at 21°C (Borowiec et al., 2015).

Except for brain, there was considerable variability among individuals in the tissue levels of HIF1α protein during hypoxia, suggesting that individual fish may be differentially impacted by low oxygen. This suggestion is supported by the observation that blood indicators of hypoxic exposure also showed considerable variation among individuals. Fish with higher blood lactate concentrations during hypoxia also had higher levels of HIF1α protein in liver tissue (Spearman's rho=0.64, *P*=0.006), although this relationship was not observed for other tissues. It is possible that variation in body mass also contributes to individual variation in HIF1α protein levels, as documented for Crucian carp (Sollid et al., 2006). In the current study, gill HIF1α protein levels in hypoxic *F. grandis* were significantly positively related to body mass (Spearman's rho=0.69, *P*=0.004), opposite of the negative relationship reported for gill HIF1α protein in normoxic and hypoxic Crucian carp (Sollid et al., 2006). Finally, *F. grandis* HIF1α protein levels were not correlated among tissues, in contrast to the positive correlations across several tissues in normoxic Crucian carp (Rissanen et al., 2006). At present, the causes and potential consequences of individual variation in tissue levels of HIF1α protein are largely unknown.

### No *HIFA* transcript changes during short-term hypoxia

Unlike HIF1α protein, *HIF1A* mRNA levels in tissues of *F. grandis* were unaffected by short-term hypoxic exposure. In other fishes, the effects of hypoxia on tissue levels of *HIF1A* mRNA vary considerably (see Introduction). In liver, *HIF1A* mRNA has been reported to increase during short-term exposure (<24 h) to levels of hypoxia similar to those used here (0.4–2.0 mg O_2_ l^−1^) in about half of the species studied, while remaining unchanged in the others (Mandic et al., 2021 and references therein). Although it is possible that some of this variation could be explained by different paralogs of *HIF1A* being measured in different studies (see Introduction), results can differ between studies measuring the same transcript within a species, using similar experimental conditions. In yellow catfish (*Pelteobagrus fulvidraco*), Wang et al. (2020) reported elevated levels of liver *HIF1Ab* mRNA after exposure to 1.14 mg O_2_ l^−1^ for 1 and 3 h, but not 6 h, whereas Pei et al. (2021) saw the opposite results, where *HIF1Ab* mRNA was unchanged at 1.5 and 4 h of exposure to 0.70 mg O_2_ l^−1^, but higher at 6.5 h. There is also disagreement among studies of fishes that express only one form of *HIF1A* (Euteleost). For example, levels of *HIF1A* mRNA were unchanged in largemouth bass (*Micropterus salmoides*) and tilapia (*Oreochomis niloticus*) exposed to levels of hypoxia similar to those used here (Yang et al., 2017; Li et al., 2017). On the other hand, Wang et al. (2017) documented elevated levels *HIF1A* mRNA in yellow croaker (*Larimichthys crocea*) at several timepoints between 1 and 24 h, even at levels of hypoxia less extreme than those used here. Although fewer studies have measured *HIF1A* mRNA in skeletal muscle and ovary, levels of this transcript were generally unaffected by hypoxia (Heinrichs-Caldas et al., 2019, Law et al., 2006, Mohindra et al., 2013), except that longer exposures (3–7 days at 1.70 mg O_2_ l^−1^) led to higher *HIF1A* mRNA in ovary of the Atlantic croaker (*Micropognias undulates*) (Rahman and Thomas, 2007).

We also measured the levels *HIF2Aa* and *HIF3A* mRNA in tissues of *F. grandis* and found no effects of acute hypoxic exposure. The limited reports on the effects of hypoxia on *HIF2A* mRNA in fishes reveal variation among tissues and species similar to *HIF1A* mRNA. Increases have been documented in gill and liver (although not in all species), but generally not in muscle or ovary (except for much longer exposures) (Geng et al., 2014; Wang et al., 2020; Rahman and Thomas, 2007). Although *HIF3A* is the least studied form, it is broadly expressed in tissues of a variety of fishes in normoxic conditions (Townley et al., 2022). This gene was originally described in the grass carp (*Ctenopharyngodon idellus*) as *HIF4A* (Law et al., 2006), but it was subsequently grouped with *HIF3A* from other ray-finned fishes (Townley et al., 2022). Law et al. (2006) exposed grass carp to 0.5 mg O_2_ l^−1^, sampled fish at 4 and 96 h, and measured levels of *HIF3A* mRNA in several tissues by northern blotting. *HIF3A* mRNA was higher than normoxic controls in all tissues at both durations of hypoxia, except skeletal muscle at 4 h.

It is important to note that we were unable to measure the various *HIFA* transcripts in *F. grandis* brain due to tissue limitation. As mentioned above, this tissue had the most robust and reproducible increase in HIF1α protein during hypoxia, and we cannot exclude the possibility that *HIF1A* also increased during hypoxia in this tissue. Among fishes, an increase in brain *HIF1Ab* has been reported in yellow catfish (Pei et al., 2021; Wang et al., 2021), but not for either *HIF1A* paralog in a variety of other species (Law et al., 2006; Shen et al., 2010; Rimoldi et al., 2012; Mohindra et al., 2013; Li et al., 2017; Yang et al., 2017). Similarly, there are relatively few reports of alterations in brain *HIF2A* and *HIF3A* transcripts in hypoxic fish (Law et al., 2006; Pei et al., 2021; Wang et al., 2021). Thus, the evidence of hypoxia-dependent changes in *HIFA* transcripts in this tissue among fishes is limited.

How can we reconcile the lack of changes in *HIF1A*, *HIF2Aa* and *HIF3A* mRNA in tissues of hypoxic *F. grandis* with reports that these transcripts increase in abundance in certain tissues during hypoxic exposure of selected species? The reasons for this difference are unknown but likely arise from biological (e.g. species, paralogs) and technical (e.g. hypoxic exposures and experimental methods) variation among studies. Also, we cannot exclude the possibility that longer hypoxic exposures cause changes in mRNA levels of *HIFA* transcripts in *F. grandis*, as proposed for *HIF2Aa* in ovary of Atlantic croaker (Rahman and Thomas, 2007). As pointed out by others, however, caution should be exercised when interpreting changes in mRNA levels because it is not certain if the corresponding protein levels increase, nor that downstream gene expression is activated (O'Brien et al., 2020; Mandic et al., 2021).

### Implications for the mechanism of HIF1α protein increases during acute hypoxia

The current study showed that short-term exposure to hypoxia led to higher mean levels of HIF1α protein in certain tissues, even when mean mRNA levels were unchanged. These results are similar to those reported for fish cells in culture, early developmental stages, and adult tissues (Soitamo et al., 2001; Law et al., 2006; Rissanen et al., 2006; Sollid et al., 2006; Robertson et al., 2014; Guan et al., 2017). We demonstrated for the first time, to our knowledge, that HIF1α protein levels do not correlate with *HIF1A* mRNA levels when measured in the same tissues among individual fish exposed to either normoxia or hypoxia. Both lines of evidence argue that new transcription is not required for the initial increase in HIF1α protein levels in tissues of *F. grandis* exposed to hypoxia. Rather, the current results support protein stabilization as the mechanism underlying the increase in HIF1α protein during short-term hypoxia as reported for mammals and fish cells in culture (Kaelin and Ratcliffe, 2008; Soitamo et al., 2001). While these conclusions align with the widely accepted mechanism of hypoxic induction of HIF1, the current study reinforces the importance of measuring HIF1α protein levels, and ultimately target gene expression, in order to better understand the molecular responses of fish to low oxygen.

## MATERIALS AND METHODS

### Fish collection and maintenance

Adult female *F. grandis* (*n*=27; mean mass, 16.29 g; range, 10.17–24.93 g) were purchased in February 2019 and housed at the University of New Orleans. Fish were treated within one week for bacterial infection and ectoparasites with API Furan-2 and API General Cure (Chalfont, PA, USA). Fish were housed in two 38-L aquaria in dechlorinated water that was adjusted to a salinity of 9–12 using Instant Ocean Synthetic Sea Salt (Blacksburg, VA, USA). Water was maintained at 24.6±1.2°C (mean±range), aerated to maintain >80% (87.3±4.4%) air saturation, and filtered. Dissolved oxygen (DO, in % saturation and mg O_2_ l^−1^), temperature, and salinity were monitored daily using a YSI Pro2030 oxygen-temperature-salinity probe (Yellow Springs, OH, USA). Nitrates, nitrites, and ammonia were measured once a week, and partial water changes (∼25%) were conducted as needed to keep these variables within acceptable levels. Fish were fed 1–1.5% of their body mass daily between 10:00 and 12:00 with TetraMin Tropical Flake fish food (Blacksburg, VA, USA) and maintained under a 12 h light:12 h dark photoperiod. Procedures for handling and experiments adhered to established guidelines approved by the University of New Orleans Institutional Animal Care and Use Committee (Protocol 18-006).

### Experimental exposures

Experimental exposures were conducted between 1 and 2 months after collection. The day before experiments, fish were fed in the morning (22 to 28 h before exposures), transferred to the exposure tank in the evening, and allowed to adjust to the tank overnight. The 76-L exposure tank was subdivided by polystyrene grate into four individual fish compartments, each of which had a removable polystyrene grate below the air-water interface to prevent fish from accessing the surface. The four compartments were separated by opaque dividers, preventing fish from seeing one another. Water in the exposure tank was thoroughly circulated throughout the tank by submersible water pumps, and it had the same composition as the maintenance tanks except for dissolved oxygen (see below).

Groups of two to four fish were randomly selected from the holding tanks and assigned to one of four treatments: 6 h normoxia; 24 h normoxia; 6 h hypoxia; 24 h hypoxia. These treatments were repeated with independent groups of fish to achieve the final sample sizes: 6 h normoxia, *n*=6; 24 h normoxia, *n*=4; 6 h hypoxia, *n*=9; 24 h hypoxia, *n*=8. Sample sizes for the hypoxic groups were intentionally larger than normoxic groups because of previous observations of inter-individual variation in levels of HIF1α protein during hypoxia (Gonzalez-Rosario, 2016). For normoxic exposures, water was continuously aerated with room air to maintain DO>7 mg O_2_ l^−1^ (∼90% air-saturation). For hypoxic exposures, nitrogen gas was bubbled into the exposure tank under the control of a CanaKit Raspberry Pi 3 Model B+ (North Vancouver, BC, Canada), which received input from a galvanic oxygen electrode (Atlas Scientific, Long Island City, NY, USA). Nitrogen was introduced to achieve the target DO of 1 mg O_2_ l^−1^ (∼13% air-saturation), which took 45–60 min, and then as needed to maintain the target. The exposure tank was continuously bubbled with air at a low rate to prevent the DO from dropping below the target. Plastic bubble wrap was placed on the surface of the water to minimize diffusion of oxygen from ambient air. Manual measurements with the YSI Pro2030 demonstrated that oxygen, temperature, and salinity were uniform throughout the exposure tank.

### Euthanasia and tissue sampling

After 6 or 24 h exposure to either normoxia or hypoxia, fish were netted and immersed in a slurry of aquarium water and ice (<2°C) until loss of equilibrium (Larter and Rees, 2017). Fish were briefly blotted and bled by severing the caudal peduncle. Blood samples (30–50 µl) were collected in heparinized capillary tubes and analyzed for indicators of hypoxic exposure (see below). Euthanasia was confirmed by severing the spinal cord behind the head, after which, fish were weighed and dissected for brain, ovaries, skeletal muscle, liver, and gills. Tissues were frozen in liquid nitrogen, placed on dry ice, and transferred within 2 h to −80°C, where they were kept until analysis (within 6 months). Tissue sampling occurred from 13:30 to 16:30.

### Blood variables

For each fish, hematocrit was determined by centrifugation of one blood sample in a microhematocrit centrifuge (BD Clay Adams AutoCrit Ultra 3, Franklin Lakes, NJ, USA) for 3 min. A second blood sample was collected and immediately diluted into 1.0 ml of saline consisting of 145 mM NaCl, 5 mM KCl, 12 mM NaHCO_3_, 3 mM NaH_2_PO_4_, with 3 mM sodium citrate (dihydrate) to prevent clotting (final pH 7.6) (Genz and Grosell, 2011; Wood et al., 2010). Red blood cells (RBC) were counted in the diluted blood using a Neubauer hemocytometer. The same diluted blood sample was frozen at −20°C, thawed, and vortexed vigorously to lyse red blood cells. Lysates were centrifuged at 16,000×***g*** for 60 s, and hemoglobin concentration was determined in the supernatant with a 96-well plate hemoglobin assay (Cayman Hemoglobin Colorimetric Assay Kit, Ann Arbor, MI, USA). RBC count (cells per ml), blood hemoglobin (Hb; g dl^−1^), and mean corpuscular hemoglobin concentration (MCHC; g Hb l^−1^ cells) were determined after accounting for the dilution of whole blood by saline.

A third blood sample was collected and prepared for blood glucose and lactate measurements as described by Larter and Rees (2017). Glucose was determined using a glucose oxidase-peroxidase coupled colorimetric assay according to the manufacturer's directions (Glucose Colorimetric Assay Kit, Ann Arbor, MI, USA). Lactate was determined as described by Virani and Rees (2000) with the following modifications for a 96-well plate format. The final assay volume was 250 µl, and contained glycine (192 mM), hydrazine (160 mM), NAD^+^ (5 mM), LDH (12 units/ml), and lactate standard or sample. Each standard and sample was assayed in quadruplicate, with an equal volume of water replacing LDH in two wells to account for non-specific absorbance. Plates were agitated for 5 s, incubated at 37°C for 1 h, and read at 340 nm using a Molecular Devices Versamax plate reader (San Jose, CA, USA).

### Tissue lysate preparation and HIF1α protein analysis

Frozen tissues were rapidly weighed, and 50–100 mg was ground under liquid nitrogen in pre-cooled mortars and pestles. Frozen tissue powder was added to 1 ml lysis buffer [137 mM NaCl, 2.7 mM KCl, 10 mM Na_2_HPO_4_, 1.8 mM KH_2_PO_4_, containing 1% Igepal, 0.5% sodium deoxycholate, 0.1% SDS, 1 mM EDTA, 1 mM sodium orthovanadate, 50 µg MG-132 ml^−1^ (Thermo Fisher Scientific, Lenexa, KS, USA) and 1% protease inhibitor cocktail (Sigma-Aldrich, St. Louis, MO, USA)] and homogenized with 2×10 strokes using Teflon-glass homogenizers (Thomas Scientific, Houston, TX, USA) on ice. Lysates were centrifuged at 4°C for 10 min at 10,000×***g***, and the supernatants were frozen at −80°C. Protein concentration was determined by the bicinchoninic acid protein assay (Smith et al., 1985) using bovine serum albumin as the standard (Pierce, ThermoFisher Scientific, Rockford, IL, USA).

HIF1α was immunoprecipitated from tissue lysates using chicken polyclonal antibodies developed against HIF1α from *F. heteroclitus* (Townley et al., 2017) and affinity-purified in Gonzalez-Rosario (2016). The affinity-purified antibody does not cross react with *in vitro* transcribed and translated *F. heteroclitus* HIF2α or HIF3α (Gonzalez-Rosario, 2016). All steps were conducted at 0–4°C unless otherwise indicated. In brief, a volume of lysate containing 1–2 mg protein (1 mg in brain; 1.25 mg in skeletal muscle; 2 mg in ovaries, liver, and gills) was brought to a final volume of 1.0 ml with lysis buffer. Reactions were cleared of non-specific binding by incubating with 25 µl of PrecipHen reagent (agarose-coupled goat anti-chicken IgY; Aves Lab; Davis, CA, USA) for 30 min with gentle rocking. Reactions were centrifuged at 1500×***g*** for 10 min. The supernatants were removed to clean tubes and incubated with affinity-purified HIF1α antibody (5 µl, equivalent to 5 µg IgY) for 1 h, after which PrecipHen reagent (25 µl) was added to each reaction and incubated with end-over-end rocking overnight (16–20 h). Precipitated antigen-antibody-PrecipHen complexes were washed sequentially in 0.5 ml each of TBS (20 mM Tris, pH 7.6, 150 mM NaCl) containing 0.05% Tween-20, TBS, and 50 mM Tris pH 6.8, centrifuging each time at 1500 ***g*** for 5 min. The final immunoprecipitates were resuspended in Laemmli sample buffer (Laemmli, 1970) containing 50 mM dithiothreitol, heated at 95°C for 3 min, clarified by centrifugation, and stored at −20°C.

Immunoprecipitated proteins were separated by sodium dodecyl sulfate polyacrylamide gel electrophoresis at 200 V for 50–60 min (Laemmli, 1970). Gels included molecular weight markers (Cell Signaling Biotinylated Protein Ladder, Danvers, MA, USA; Novex Sharp Pre-stained Protein Standard, Waltham, MA, USA) and positive HIF1α controls (*in vitro* transcribed and translated *F. heteroclitus* HIF1α; Townley et al., 2017). After electrophoresis, proteins were transferred to polyvinylidene difluoride membranes by electrotransfer at 100 V for 1 h at 10°C in 25 mM Tris, 192 mM glycine, 20% methanol, 0.05% SDS (Towbin et al., 1979). The efficiency of electrotransfer was verified by the absence of proteins in gels stained by colloidal Coomassie blue (Neuhoff et al., 1988). Blots were blocked in TBS containing 0.05% Tween-20 (TBS-T) and 5% non-fat dry milk at room temperature for 1 h, followed by incubation in the same buffer containing 1:500 dilution of chicken anti-HIF1α. After overnight incubation in primary antibody at 4°C, blots were washed with TBS-T three times and then incubated in TBS-T containing 5% non-fat dry milk and anti-biotin horseradish peroxidase (Cell Signaling, Danvers, MA, USA) and HRP-conjugated donkey anti-chicken antibody (Sigma-Aldrich, St. Louis, MO, USA) diluted to 1:2000 and 1:5000, respectively. Blots were washed with TBS-T five times, developed in enhanced chemiluminescent detection reagents (Harlow and Lane, 1988) at room temperature for 60 s, and imaged with a ChemiDoc MP (Bio-Rad, Hercules, CA, USA). Protein band intensities were determined with ImageLab (Bio-Rad) using automatic background subtraction. Because of the specificity of the HIF1α antibody used in immunoprecipitation, it was not possible to probe the same blots for a ‘housekeeping’ protein for normalization purposes. To account for differences in immunoprecipitation efficiency across samples, the relative HIF1α protein abundance was determined by dividing the HIF1α band intensity by the intensity of the immunoprecipitating IgY band for the same sample (see Results).

### mRNA preparation and analysis

Frozen samples of skeletal muscle, liver, ovary, and gill (approximately 15–20 mg each) were ground under liquid nitrogen in pre-cooled mortars and pestles, and extracted for RNA using commercial kits (RNeasy fibrous tissue kit, Qiagen, Valencia, CA, USA). The manufacturer-supplied protocol was modified to include a second round of DNase I treatment in solution to ensure no genomic contamination. The concentration of RNA was measured using a NanoDrop ND-1000 spectrophotometer (NanoDrop Technologies, Wilmington, DE, USA). All samples had A260:A280 ratios between 1.8 and 2.2, indicating high RNA purity. RNA integrity was determined using a 2100 Bioanalyzer (Agilent, Santa Clara, CA, USA), and only samples with RNA integrity numbers greater than 7.3 were used in subsequent steps. Total RNA was diluted to final concentrations of 500 ng µl^−1^ for ovary and muscle samples or 1 µg µl^−1^ for liver and gill, and RNA contained in 1 µl was reverse transcribed by TaqMan reverse transcriptase with random hexamer primers (Applied Biosystems, Foster City, CA, USA). Samples lacking the reverse transcriptase were prepared simultaneously as controls to ensure the absence of genomic DNA.

Quantitative real time PCR (qPCR) for *HIF1A*, *HIF2Aa*, *HIF3A*, and *ARNT2* was carried out using an iQ5 real-time PCR detection system (Bio-Rad) and gene-specific primers (Townley et al., 2017). Each qPCR reaction was performed in 25 µl reactions which contained 12.5 µl SYBR Green PCR Master Mix (Applied Biosystems), 0.1 µM of each primer, and 1 µl of cDNA. The qPCR protocol followed a denaturation step for 20 s at 90°C, then 40 cycles of 95°C for 10 s and 60°C for 30 s. Melting curves were obtained by increasing temperature from 50 to 95°C in 90 steps of 0.5°C to ensure the presence of a single amplicon. All qPCR assays were performed in duplicate except when the standard deviation between the duplicates was more than 0.5 cycle, in which case, assays were done in quadruplicate. A pooled cDNA sample was formed by combining equal volumes of cDNA from at least six individuals per treatment group. This pooled sample was serially diluted to assess primer efficiencies, which ranged from 90–105%. The cDNA pooled sample was also included in every 96-well plate for a given gene to correct for slight variation in threshold cycle number for the same sample across multiple runs for a given gene. Finally, several samples were randomly selected and subjected to PCR for each gene without prior treatment with reverse transcriptase. These samples had threshold cycle numbers similar to water, which was included in every plate, and confirmed that genomic DNA was not amplified.

Relative mRNA abundance was computed according to Pfaffl (2001), which calculates mRNA levels relative to a control treatment and levels of a control gene, after accounting for the amplification efficiencies for different genes. For this calculation, the average threshold cycle number of all normoxic samples (6 and 24 h) for a given gene was used as the control against which each sample for that gene was compared. To control for variation in RNA among samples, the threshold cycle number for all *HIFA* transcripts were normalized by the threshold cycle number for *ARNT2*, the major teleost-specific *ARNT* paralog in fishes (Powell et al., 1999), which previous research has shown to be unaffected by hypoxic exposure (Rahman and Thomas, 2019).

### Statistical analyses

Normality of response variables was determined using Shapiro–Wilk tests, and equality of group variances was tested using Bartlett's tests. Variables that were not normally distributed were transformed using the Box–Cox technique (Box and Cox, 1964). The effects of hypoxia treatment, exposure time, and the interaction between treatment and time were assessed by two-way analysis of variance (ANOVA). Relationships between selected response variables were assessed by Spearman rank order correlation, and false-discovery rate corrections were performed using the R package (p.adjust). Statistical analyses were performed in R version 3.6.1 (R Studio Team, 2022) and graphs were created in GraphPad Prism version 7.01 (GraphPad Software, Boston, MA, USA).

## Supplementary Material

10.1242/biolopen.060167_sup1 Supplementary information
