# Supplementary material for: Hypoxia-inducible factor 1 alpha protein increases without changes in mRNA during acute hypoxic exposure of the Gulf killifish, Fundulus grandis
Source: Biol Open. 2023 Dec 27;12(12):bio060167. doi: 10.1242/bio.060167 (PMC10805151; doi:10.1242/bio.060167)
Supplement: Supplementary information [file biolopen-12-060167-s1.pdf]

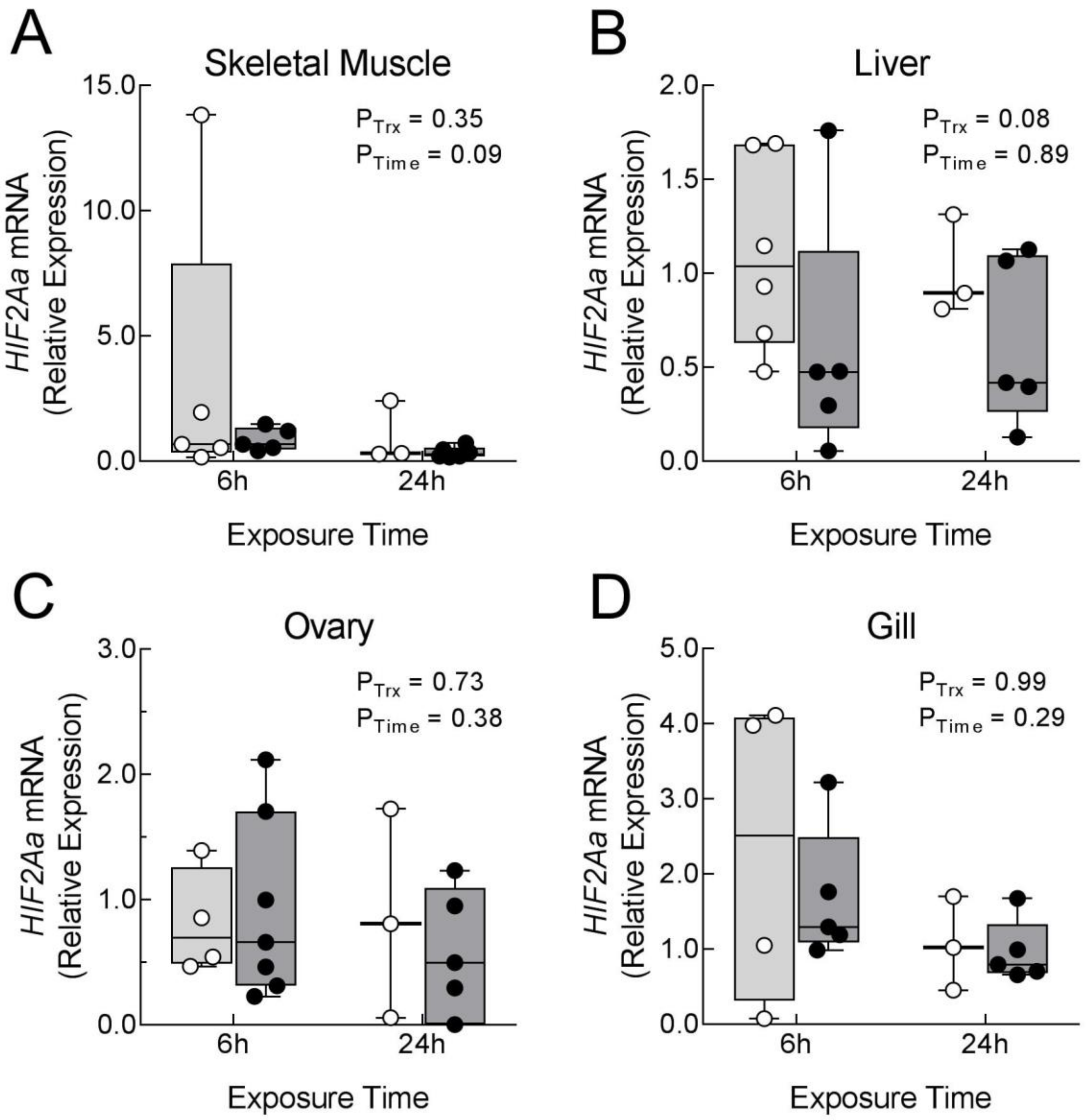

Fig. S1. ***HIF2a*** **mRNA levels in tissues of** ***Fundulus grandis*** **during acute hypoxia.** Levels of *HIF2a* mRNA were determined by qPCR and expressed relative to the level of *ARNT2* mRNA in the same samples. *HIF2a* mRNA abundance was determined for skeletal muscle (A), liver (B), ovary (C), and gill (D) after exposure of fish to normoxia (> 7 mg O_2 l^{-1}; open symbols and light grey boxes) or hypoxia (~1 mg O_2 l^{-1}; filled symbols and dark grey boxes) for 6 or 24 h. Lines of box and whiskers plots represent (from lowest to highest) the minimum, the 25th percentile, the median, the 75th percentile, and the maximum. The effects of hypoxia treatment (P_{Trx}), exposure time (P_{Time}), and the interaction between treatment and time were determined by 2-way ANOVA (all interactions were non-significant and not shown).

Biology Open • Supplementary information

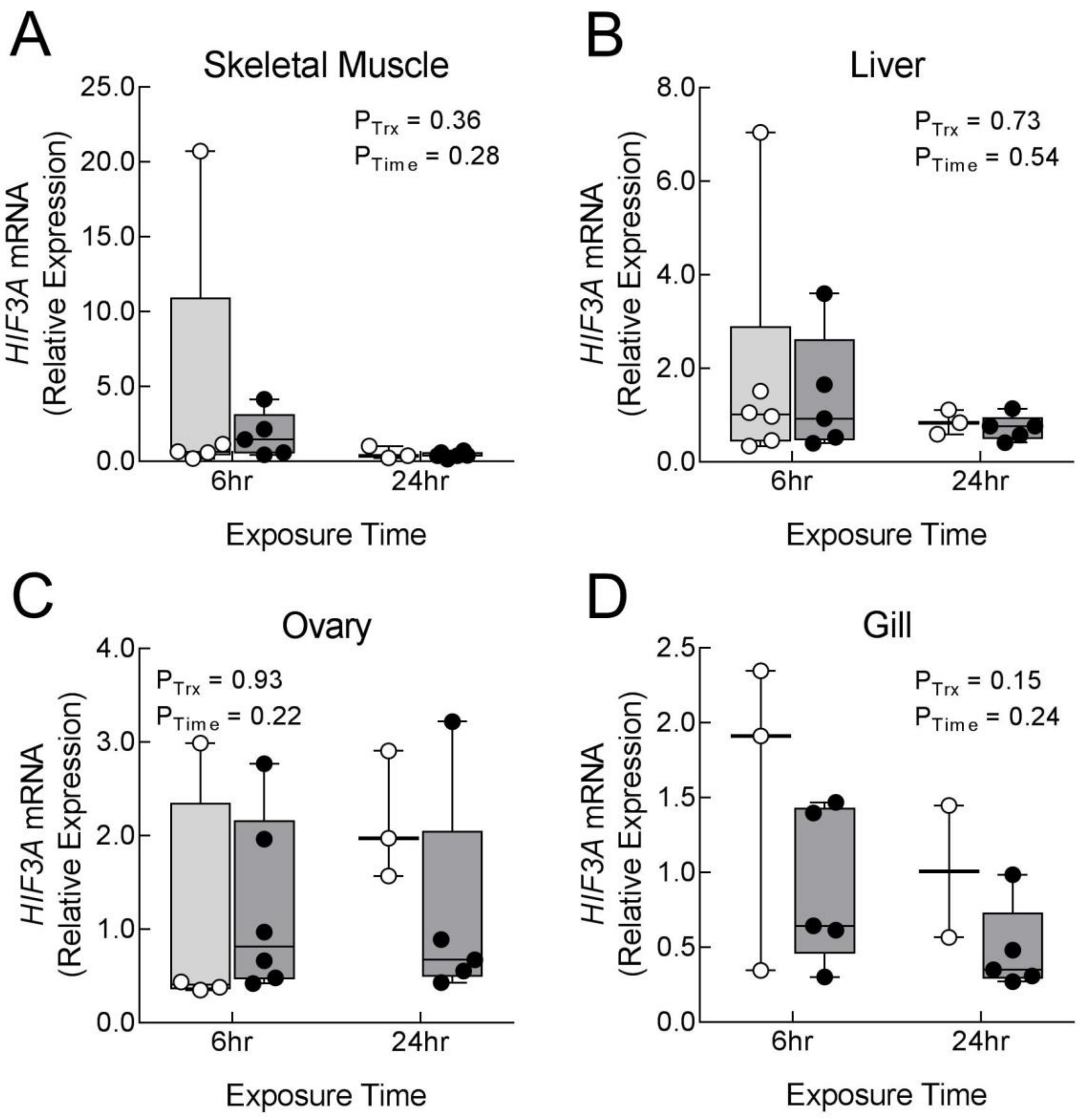

Fig. S2. *HIF3A* mRNA levels in tissues of *Fundulus grandis* during acute hypoxia. Levels of *HIF3A* mRNA were determined by qPCR and expressed relative to the level of *ARNT2* mRNA in the same samples. *HIF3A* mRNA abundance was determined for skeletal muscle (A), liver (B), ovary (C), and gill (D) after exposure of fish to normoxia (> 7 mg O_2 l^{-1}; open symbols and light grey boxes) or hypoxia (~1 mg O_2 l^{-1}; filled symbols and dark grey boxes) for 6 or 24 h. Lines of box and whiskers plots represent (from lowest to highest) the minimum, the 25th percentile, the median, the 75th percentile, and the maximum. The effects of hypoxia treatment (P_{Trx}), exposure time (P_{Time}), and the interaction between treatment and time were determined by 2-way ANOVA (all interactions were non-significant and not shown).

Biology Open • Supplementary information

Table S1. Correlation analyses of HIF1α protein levels among tissues of *Fundulus grandis* after laboratory exposure to normoxia (>7 mg O_2 l^{-1}) or hypoxia (~1 mg O_2 l^{-1}).

Normoxia	Brain	Muscle	Liver	Ovary	Gill
Brain	---	0.10 (8)	0.58 (9)	0.43 (8)	0.51 (8)
Muscle	0.93	---	-0.07 (8)	-0.04 (8)	-0.33 (8)
Liver	0.50	0.93	---	0.66 (9)	0.44 (8)
Ovary	0.58	0.93	0.50	---	-0.12 (8)
Gill	0.58	0.70	0.58	0.93	---

Hypoxia	Brain	Muscle	Liver	Ovary	Gill
Brain	---	0.37 (12)	0.14 (13)	0.43 (11)	0.10 (11)
Muscle	0.40	---	0.31 (16)	-0.23 (14)	-0.37 (14)
Liver	0.71	0.40	---	-0.38 (14)	-0.13 (15)
Ovary	0.40	0.61	0.40	---	0.48 (12)
Gill	0.77	0.40	0.71	0.40	---

For each sample, HIF1α protein was expressed as the band intensity in western blots relative to the intensity of IgY band in the same sample (see Materials and Methods). Correlations between pairs of tissues were assessed by Spearman’s rank order correlation (rho), shown above the diagonal, with the corresponding probabilities (*P*), shown below the diagonal. *P* values were adjusted for false discovery. Separate analyses were done on normoxic and hypoxic samples, pooling 6 h and 24 h timepoints within each treatment. Sample sizes are shown in parentheses.

Table S2. Correlation analyses of *HIF1A* mRNA levels among tissues of *Fundulus grandis* after exposure to normoxia (>7 mg O_2 l^{-1}) or hypoxia (~1 mg O_2 l^{-1}).

Normoxia	Muscle	Liver	Ovary	Gill
Muscle	---	-0.40(8)	-0.07 (7)	-0.60 (5)
Liver	0.32	---	0.29 (7)	0.14 (6)
Ovary	0.88	0.53	---	-0.60 (5)
Gill	0.28	0.79	0.28	---

Hypoxia	Muscle	Liver	Ovary	Gill
Muscle	---	0.14 (7)	0.02 (10)	0.20 (6)
Liver	0.78	---	-0.12 (8)	-0.50 (7)
Ovary	0.96	0.78	---	-0.14 (7)
Gill	0.70	0.25	0.76	---

For each sample, *HIF1A* mRNA was expressed relative to the level of *ARNT2* mRNA in the same sample (see Materials and Methods). Correlations between pairs of tissues were assessed by Spearman's rank order correlation (rho), shown above the diagonal, with the corresponding probability of a significant relationship (*P*), shown below the diagonal. *P* values were adjusted for false discovery. Separate analyses were done on normoxic and hypoxic samples, pooling 6 h and 24 h timepoints within each treatment. Sample sizes are shown in parentheses.

Biology Open • Supplementary information

Table S3. Copy number for *HIF1A*, *HIF2Aa*, *HIF3A*, and *ARNT2* from *Fundulus grandis* tissues after 6 or 24 h of normoxia (Norm) or hypoxia (Hyp).

Gene	Tissue	6 h Norm	24 h Norm	6 h Hyp	24 h Hyp	P_{trx}	P_{time}	P_{intrxn}
HIF1A	Muscle	306 ± 185 (n=5)	545 ± 414 (n=3)	391 ± 212 (n=5)	320 ± 292 (n=6)	0.81	0.81	0.67
	Liver	255 ± 274 (n=6)	318 ± 192 (n=3)	188 ± 164 (n=7)	200 ± 90 (n=6)	0.42	0.28	0.27
	Ovary	177 ± 88 (n=4)	562 ± 89 (n=3)	790 ± 1097 (n=7)	597 ± 383 (n=5)	0.77	0.73	0.10
	Gill	309 ± 97 (n=4)	370 ± 297 (n=3)	471 ± 410 (n=3)	1616 ± 1190 (n=5)	0.09	0.09	0.22
HIF2Aa	Muscle	3566 ± 1747 (n=5)	3413 ± 3503 (n=3)	4925 ± 2620 (n=5)	4002 ± 4406 (n=6)	0.45	0.49	0.97
	Liver	1899 ± 822 (n=6)	4075 ± 3035 (n=3)	1397 ± 1478 (n=7)	2896 ± 1687 (n=6)	0.23	0.02	0.20
	Ovary	1534 ± 518 (n=4)	8569 ± 8161 (n=3)	5723 ± 7531 (n=7)	3999 ± 3010 (n=5)	0.55	0.89	0.15
	Gill	31251 ± 20513 (n=4)	29375 ± 30788 (n=3)	37297 ± 21672 (n=5)	76049 ± 42341 (n=5)	0.19	0.15	0.32
HIF3A	Muscle	25 ± 20 (n=5)	27 ± 32 (n=3)	46 ± 21 (n=5)	27 ± 28 (n=6)	0.19	0.46	0.71
	Liver	17 ± 18 (n=6)	33 ± 38 (n=3)	24 ± 21 (n=7)	28 ± 22 (n=6)	0.13	0.12	0.22
	Ovary	12 ± 6 (n=4)	29 ± 17 (n=3)	182 ± 253 (n=7)	80 ± 60 (n=5)	0.92	0.81	0.16
	Gill	30 ± 12 (n=3)	44 ± 58 (n=2)	52 ± 48 (n=5)	86 ± 45 (n=5)	0.19	0.25	0.69
ARNT2	Muscle	168 ± 194 (n=5)	365 ± 327 (n=3)	216 ± 129 (n=5)	333 ± 278 (n=5)	0.38	0.31	0.75
	Liver	19 ± 9 (n=6)	40 ± 35 (n=3)	30 ± 31 (n=5)	52 ± 47 (n=6)	0.17	0.14	0.23
	Ovary	689 ± 357 (n=4)	1872 ± 2462 (n=3)	2091 ± 1984 (n=5)	1616 ± 959 (n=5)	0.29	0.69	0.23
	Gill	187 ± 233 (n=3)	328 ± 278 (n=3)	212 ± 186 (n=6)	858 ± 565 (n=5)	0.16	0.07	0.29

Values are reported as mean (± S.D.) transcript number per 50 ng total RNA. Results of two-way ANOVAs on Box-Cox transformed copy number are shown with probability values for the effects of treatment (P_{trx}), time (P_{time}), and their interaction (P_{intrxn}). Sample sizes (n) are shown in parentheses.

Biology Open • Supplementary information
